# The Association of Infant Birth Sizes and Anemia under Five Years Old: A Population-Based Prospective Cohort Study in China

**DOI:** 10.3390/nu16121796

**Published:** 2024-06-07

**Authors:** Xiaojing Liu, Xiaowen Liu, Zeping Yang, Zhiwen Li, Le Zhang, Yali Zhang, Jianmeng Liu, Rongwei Ye, Nan Li

**Affiliations:** 1 Department of Epidemiology and Biostatistics, School of Public Health, Peking University Health Science Center, Beijing 100191, China; liuxiaojing@bjmu.edu.cn (X.L.); lxw524@pku.edu.cn (X.L.); yangzeping1028@pku.edu.cn (Z.Y.); lizw@bjmu.edu.cn (Z.L.); zhangle@bjmu.edu.cn (L.Z.); zhangyl@bjmu.edu.cn (Y.Z.); liujm@pku.edu.cn (J.L.); yerw@bjmu.edu.cn (R.Y.); 2 Institute of Reproductive and Child Health/Ministry of Health Key Laboratory of Reproductive Health, Peking University Health Science Center, Beijing 100191, China

**Keywords:** infant birth size, childhood anemia, birth weight, crown–heel length, head circumference

## Abstract

Infant birth sizes are vital clinical parameters to predict poor growth and micronutrient deficiency in early life. However, their effects on childhood anemia remain unclear. We aimed to explore the associations between birth weight, crown–heel length, and head circumference with anemia in early childhood, as well as potential modification factors. This population-based prospective cohort study included 204,556 participants with singleton live births delivered at gestational ages of 28–42 weeks. A logistic regression model was used to estimate the associations of the measures of infant birth size and their Z-score with anemia under five years old. There were 26,802 (13.10%) children under five years old who were diagnosed has having anemia. Compared with children who did not have anemia, children who had anemia had a lower birth weight and smaller head circumference and a longer crown–heel length (all *p*-values < 0.05). After adjusting for confounders, not only birth weight (β coefficient, −0.008; 95% CI, −0.011–−0.004; *p* < 0.001) and head circumference (β coefficient, −0.004; 95% CI, −0.007–−0.001; *p* = 0.009), but also the related Z-scores were negatively associated with childhood anemia, while the trends for crown–heel length were the opposite. We further found significant interactions of folic acid use and maternal occupation with infant birth sizes. In conclusion, infants having abnormal sizes at birth are significantly associated with the risk for childhood anemia, which can be modified by folic acid use during pregnancy and maternal occupation.

## 1. Introduction

Children having anemia comprise a major public health concern worldwide, with approximately 300 million children under 5 years old diagnosed as having anemia [1]. Particularly in low- and middle-income countries, the prevalence of anemia among children aged 6 to 59 months is greater than 40% [2,3]. Childhood anemia continues to be a severe issue associated with an increased risk of childhood mortality; also, children who have anemia have a high likelihood of impaired cognitive development, and anemia leads to long-term health complications [4,5,6]. As the largest developing country, China is projected to achieve a target for reducing anemia prevalence to less than 10% in children who are young. However, there remains a great gap in our knowledge regarding the risk factors for childhood anemia, especially those that originate from the prenatal period.

Infant birth sizes, including body weight, crown–heel length, and head circumference at birth, are widely used to evaluate intrauterine growth and nutrition status [7]. Low birth weight after abnormal fetal growth is of prognostic significance for predicting perinatal death, congenital malformations, or nervous system abnormalities in early life [8,9,10]. Notably, infant sizes at birth have a linear relation with the amount of iron stores during the first 6–8 months of life [11,12]. There is conclusive evidence that iron deficiency can impair red blood production and result in a significant decrease of hemoglobin concentration [13]. Thus, infant birth sizes may be implicated in childhood anemia status via affecting iron stores in early infancy.

Our previous studies have indicated that maternal and fetal complications are significantly associated with an increased risk of offspring that have anemia [14,15]. Those pregnancy complications are leading causes of fetal malnutrition and intrauterine growth restriction, which indicate that neonates are more likely to be born having abnormal sizes [16]. Herein, we speculated that infant sizes at birth might correlate with the development of childhood anemia. The present study aimed to investigate their associations and potential modified factors with a large and prospective birth cohort study in China.

## 2. Methods

### 2.1. Cohort Background and Study Population

**Figure 1:**
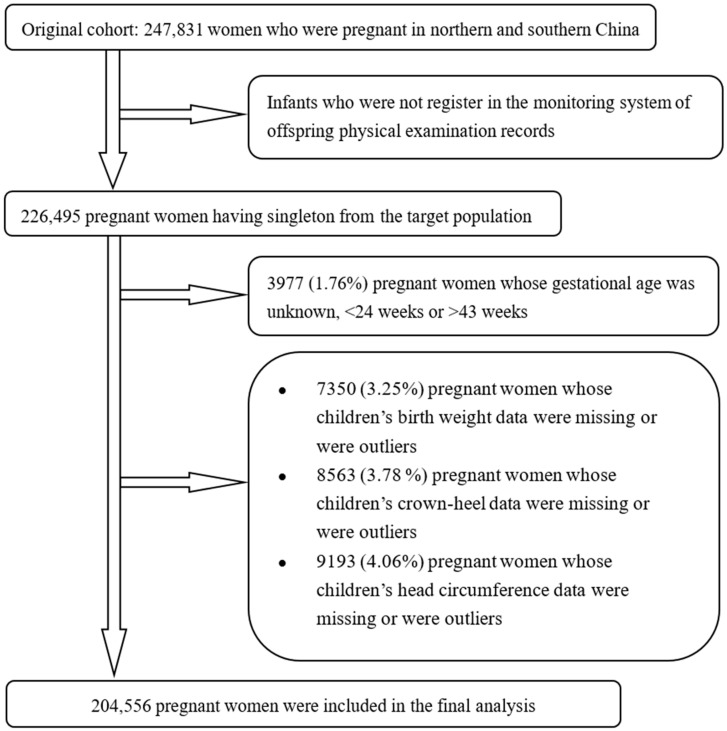
Flowchart of participants in the study.

The present study was derived from a large population-based prospective cohort that has been described in previously published literature [17,18]. Beginning in October 1993, a campaign for neural-tube defect prevention was conducted in rural regions from one northern province (Hebei) and two southern provinces of China. All women who were pregnant and women who were preparing for marriage registered in a perinatal healthcare surveillance system. The population was followed up to measure the anthropometric data and hemoglobin concentrations of infants who were under five years old, aiming to evaluate the effect of taking folic acid on the maternal and infant health outcomes. We identified 226,495 women who had singleton births who were pregnant with the data from offspring physical examination records in the perinatal healthcare surveillance system. We excluded 3977 women who were pregnant (1.76%) whose gestational age was unknown, <24 weeks, or >43 weeks. We further excluded 7350 (3.25%), 8563 (3.78%), 9193 (4.06%), and 9521 (4.20%) women who were pregnant whose children’s data regarding birth weight, crown–heel length, head circumference, and hemoglobin concentration were unknown or were outliers, respectively. After those exclusions, we included 204,556 women who were pregnant (90.31% of the target population) in the final analysis (Figure 1).

### 2.2. Infant Birth Size

Infant birth sizes in the present study were characterized by neonatal birth weight in grams (g) and crown–heel length and head circumference in millimeter (mm), which were measured by a professional workers who work in health within the first hour after delivery. A neonate who was naked had his/her body weight measured in 10 g increments. All instruments were well-calibrated by local authorities who ensured their quality and technical supervision. The detailed data on the neonatal anthropometric measurements were collected via linking to the perinatal healthcare surveillance system. Furthermore, the measures of infant birth size were standardized using the Z-score. For each week of gestational age (GA) at birth, the Z-scores for birth weight, crown–heel length, and head circumference were assigned to each participant on the basis of GA-specific distributions of Chinese newborns from 1998–2000 [19]. Then, the standard deviation score (SDS) for the measures of infant birth size and their Z-score was calculated based on the corresponding mean value and 0.5 standard deviation (SD) of the study population.

### 2.3. Definition of Anemia

A detailed description regarding hemoglobin measurement has been stated in our previous study [15]. We used a standard cyanmethemoglobin method to detect hemoglobin concentration in capillary blood with a HemoCue system (HemoCue AB, Angelholm, Sweden) and a hemoglobinometer (model 721, Shanghai, China). To minimize measurement bias, the doctors were well-trained and the room temperature was maintained above 18 °C. According to the WHO guidelines, anemia is defined as hemoglobin concentrations < 110 g/L for infants and children who are aged < 60 months [20].

### 2.4. Covariates

Demographic information at baseline and healthcare records during follow-up were acquired from the perinatal healthcare surveillance system. The body mass index (BMI) was calculated by weight in kilograms divided by the square of height in meters (kg/m^2^). Anemia during pregnancy was defined as maternal hemoglobin concentration < 110 g/L at any time during pregnancy. Women who took folic acid alone at any time from the registration period until the end of the first trimester of pregnancy were defined as users of folic acid. Categorical variables were classified as follows: ethnicity (Han or other), education (high school or higher, junior high school, primary school or lower, or unknown), folic acid use (yes or no), occupation (farmer or non-farmer [workers who work in factories worker, businessperson, teacher, day laborer, civil servant, or unknown]), and feeding pattern (exclusive breastfeeding or others).

### 2.5. Statistical Analyses

Continuous variables (maternal age, BMI, follow-up age, and birth sizes) are presented as the mean ± SD and categorical variables as the number and proportion. The characteristics of mothers and children were compared by childhood anemia status using Student’s *t*-test for continuous variables and the χ^2^ test for categorical variables, respectively. The logistic regression model was used to estimate the association between the measures of infant birth size and their Z-scores with the risk of childhood anemia under five years old. The regression results are reported as β coefficients corresponding to a 0.5-SD change of birth weight, crown–heel length, head circumference, and the related Z-score. The multivariate regression adjusted for the potential confounders including age, BMI, parity, ethnicity, education, anemia during pregnancy, occupation, folic acid use, follow-up age, exclusive breastfeeding, infant sex, SGA, gestational age, and caesarean delivery. The descriptive analysis results of some peri-/neonatal confounders are shown in Table S1. Subsequently, we used the stratified analysis to test the effects of potential modification (maternal occupation and folic acid use) on childhood anemia in the multivariable logistic regression model. All two-sided *p*-values of less than 0.05 were considered statistically significant. All analyses were performed with the SPSS ver. 20.0 software (SPSS Inc., Chicago, IL, USA).

## 3. Results

Among 204,556 mother–child pairs in the cohort, there were 26,802 (13.10%) children having anemia with an average follow-up age of 54.37 ± 8.06 months. The characteristics by childhood anemic status are shown in Table 1. We found that the characteristics of ethnicity and exclusive breastfeeding were comparable between the group having anemia and that not having anemia. The mothers who delivered children having anemia were more likely to be older, be thinner, be farmers, have a lower BMI, be highly educated, take folic acid supplements, and have anemia during pregnancy when compared with those delivering children who did not have anemia.

We compared the differences of infant birth size between the group having anemia and the group not having anemia (Table 2). The mean ± SD values of birth weight (*p* < 0.001) and head circumference (*p* = 0.043) in the group having anemia were 3296.49 ± 406.07 g and 335.14 ± 15.84 mm, significantly lower than those in the group not having anemia, which were 3306.99 ± 409.67 g and 335.35 ± 15.89 mm, respectively. Meanwhile, there were significant differences in the Z-scores for birth weight (*p* < 0.001) and head circumference (*p* < 0.001) between the two groups. However, both crown–heel length (*p* < 0.001) and the related Z-score (*p* = 0.004) showed significantly opposite trends; children having anemia had a longer crown–heel length (496.30 ± 20.04 mm) compared with children not having anemia (495.56 ± 20.18 mm).

The effects of infant birth sizes on childhood anemia are shown in Table 3. The β coefficients represent the degree of change in childhood anemia for a 0.5-SD increase in the measures of infant birth size and their Z-scores. Infant birth size had significant associations with childhood anemia with or without adjusting for the potential confounders. In the multivariate regression models, not only birth weight (β coefficient −0.008, 95% CI −0.011–−0.004, *p* < 0.001) and head circumference (−0.004, −0.007–−0.001, *p* = 0.009), but also their Z-scores were negatively associated with childhood anemia, while the association for crown–heel length was the opposite (SDS: 0.008, 0.005–0.011, *p* < 0.001; Z-score SDS: 0.008, 0.005–0.012, *p* < 0.001).

We further used stratification analysis to explore the modified effects of folic acid use and maternal occupation (Table 4). Folic acid use and maternal occupation had significant interaction relationships with birth weight, crown–heel length, and head circumference on the effects of childhood anemia. After adjusting for the potential confounders, the negative associations between birth weight and children anemia (birth weight SDS: β coefficient −0.009, 95% CI −0.014–−0.004; Z-score SDS: β coefficient −0.008, 95% CI −0.013–−0.003) in the subgroup who used folic acid were stronger than those for the subgroup who did not use folic acid (birth weight SDS: −0.007, −0.012–−0.002; Z-score SDS: −0.006, −0.011–−0.001), while similar β coefficients were found in the subgroups who were farmers and non-farmers. Among users of folic acid and those who were farmers, the positive associations of crown–heel length and childhood anemia were stronger than those who did not use folic acid; even the negative associations of head circumference were the opposite among users who did not use folic acid and those who were non-farmers.

## 4. Discussion

In this large prospective birth cohort study in China, children having anemia who were under five years old had a significantly lower birth weight, smaller head circumference, and longer crown–heel length compared with children who did not have anemia. We further found that there were significant interactions between infant birth size with folic acid use and maternal occupation on the effect of childhood anemia. As far as we are concerned, this study provides the first evidence on the associations of infant birth sizes and anemia in early childhood, as well as the potential modified effects of folic acid use and maternal occupation on those associations.

There have been limited studies about the effects of birth sizes on offspring having anemia. A low birth weight, as a marker of fetal growth restriction, correlates with severe nutritional deficit during the first weeks of life and then leads to a higher risk of long-term nutritional diseases [21]. Our previous study found that infants having a small-for-gestational-age size are associated with an increased risk of anemia at 6 months (adjusted odds ratio [aOR], 1.52; 95% CI, 1.24–1.86), 12 months (aOR, 1.42; 95% CI, 1.13–1.79), and 55 months (aOR, 1.11; 95% CI, 1.05–1.17) [14]. One cross-sectional study in a rural area of northern China also verified an association of low birth weight and infants having anemia during the first 0–18 months (aOR, 2.89; 95% CI, 1.45–5.76) [22]. Another study conducted in India between 2015 and 2016 confirmed that infants having a birth weight less than 1500 g had significantly increased risk of anemia in children who were aged 6–59 months compared with those having a normal weight (aOR, 1.48; 95% CI, 1.20–1.83) [23]. In the current study, we conducted a related analysis with birth weight as a continuous variable, and the findings supplemented additional evidence on a linear association between birth weight and childhood anemia. However, there remains a great knowledge gap about whether newborns’ crown–heel length and head circumference affect anemia development in later life. Those anthropometric parameters have been identified as important clinical signs of infants having hemoglobin deficiency, which can persist from the first year of life to early childhood [24,25,26]. The significant associations between crown–heel length (positive) and head circumference (negative) with childhood anemia in our study seem reasonable. We also elucidated the modified effects of folic acid use and maternal occupation on childhood anemia due to their interaction relationship with infant birth sizes. Together with the previous research [27], our findings provide referable value in terms of improving perinatal monitoring to identify mothers and offspring who have a high risk and for better prediction and prevention of childhood anemia.

Several potential mechanisms might be involved in the association between infant birth sizes and subsequent childhood anemia. Iron deficiency is one of the greatest causes of anemia. It has been confirmed that there is a linear association between infant birth sizes and iron status in early life [11,12]. Fetuses and neonates having abnormal birth sizes would probably have an insufficient iron store in the organs, and develop iron deficiency anemia in later life [28,29]. The increased inflammation response is another possible explanation for this association. Inflammation is a prevalent comorbidity in patients having anemia with the pathophysiology of inflammatory hypoferremia and iron-restricted erythropoiesis [30]. A recent research work has demonstrated that dysregulated inflammation increases the risk for preterm birth and restricted fetal growth; high levels of inflammatory cytokines may predict offspring having a reduced birth weight and length [31]. In addition, there are several overlapping risk factors for abnormal birth sizes and anemia, such as maternal BMI [32], nutritional status, and pregnancy complications [33,34]. Those underlying mechanisms might be well-recognized explanations for the biological link, which need more theoretical research to prove them.

There are several strengths of this study. Our findings were based on a population-based longitudinal cohort campaign, which had relatively comprehensive information of mother–child demographic characteristics. Neonatal anthropometric measurements and hemoglobin concentrations were determined by experienced workers who work in health using standardized procedures, and we obtained those data through linking to the perinatal healthcare surveillance system, thus minimizing the risk of recall bias. Additionally, the substantial sample size in this study enabled us to perform stratification analysis and reveal the modified effects of folic acid use and maternal occupation.

Some limitations should be acknowledged when interpreting our findings. Firstly, this large cohort study was prospectively conducted 20 years ago, when the sociodemographic characteristics, dietary patterns, and lifestyle of the included population might have been different from those in the contemporary period. Secondly, some maternal confounders such as smoking status and alcohol drinking were not collected in the original study. However, during the timeframe of our study, instances of smoking or alcohol consumption were relatively infrequent, particularly among women who were of reproductive-age in more agrarian Chinese settings [35]. Potential influencing factors after childbirth were also absent, e.g., length and type of nursing, dietary status, lead exposure, growth rate, and supplement intake [36,37,38]. Thirdly, the prevalence of intrauterine growth retardation (IUGR) was 0.14% in the present study, which was extremely lower than that (5.6%) obtained from a large cross-sectional survey of Chinese newborns in 1997 [39]. Thus, we did not adjust for IUGR in the multivariate analysis. Fourthly, iron status and related indexes such as serum ferritin and transferrin were unknown. Thus, we could not identify anemia subtypes and then analyze their associations with infant birth size. Finally, nearly all participants (>99%) were of Han ethnicity. The findings from this study might not apply to other ethnic groups in China. These issues should be given priority to reduce possible biases in future research.

## 5. Conclusions

In conclusion, infants with abnormal sizes at birth had significant associations with having anemia in early childhood, which can be modified by folic acid use and occupation. The findings support the importance of increased perinatal surveillance for maternal and neonatal health to enable early identification of the risk of childhood anemia. In the future, there is an urgent need for understanding the mechanism of childhood anemia associated with infant birth sizes, to inform the development of predictive and diagnostic tools and enhance therapeutic interventions of hemoglobin-associated nutrition diseases in early life.

## Figures and Tables

**Table 1:** Maternal and child characteristics according to anemic status under five years old.

Characteristics	Group Having Anemia (N = 26,802)		Group Not Having Anemia (N = 177,754)		*p*-Value
	*n*	%	*n*	%	
Mothers					
Age (years, mean [SD])	25.03 [3.51]		24.89 [3.66]		<0.001
BMI (kg/m^2^, mean [SD])	20.50 [2.00]		20.56 [2.20]		<0.001
Han ethnicity (yes)	26,604	99.39	176,352	99.35	0.458
Education					<0.001
High school or higher	8159	30.44	48,644	27.37	
Junior high school	16,065	59.94	110,614	62.21	
Primary school or lower, or unknown	2578	9.26	18,496	10.41	
Occupation					0.029
Farmer	17,148	63.98	112,499	63.29	
Non-farmer ^a^	9654	36.02	65,255	36.71	
Folic acid use (yes)	16,211	59.79	95,780	53.01	<0.001
Anemia during pregnancy (yes)	17,941	66.94	100,944	56.79	<0.001
Exclusive breastfeeding (yes)	23,738	88.57	157,006	88.33	0.253
Children					
Follow-up age (months, mean [SD])	54.37 [8.06]		55.51 [8.21]		<0.001

Abbreviations: SD, standard deviation; BMI, body mass index. ^a^ Including workers who work in factories, businessperson, teacher, day laborer, civil servant, or unknown.

**Table 2:** Differences in infant sizes at birth for children having anemia and not having anemia.

Infant Birth Sizes	Group Having Anemia (N = 26,802)	Group Not Having Anemia (N = 177,754)	*p*-Value
Body weight (g)	3296.49 (406.07)	3306.99 (409.67)	<0.001
Body weight Z-score	0.0814 (1.144)	0.127 (1.135)	<0.001
Crown–heel length (mm)	496.30 (20.04)	495.56 (20.18)	<0.001
Crown–heel length Z-score	−0.355 (1.212)	−0.377 (1.224)	0.004
Head circumference (mm)	335.14 (15.84)	335.35 (15.89)	0.043
Head circumference Z-score	−0.579 (1.408)	−0.544 (1.408)	<0.001

Infant birth sizes are presented as the mean (standard deviation).

**Table 3:** Associations of infant weight, length, and head circumference at birth on childhood anemia.

Infant Birth Sizes	Unadjusted Model			Adjusted Model ^a^		
	Coefficient	95% CIs	*p*-Value	Coefficient	95% CIs	*p*-Value
Body weight SDS	−0.006	−0.009, −0.003	<0.001	−0.008	−0.011, −0.004	<0.001
Body weight Z-score SDS	−0.009	−0.012, −0.006	<0.001	−0.007	−0.010, −0.003	<0.001
Crown–heel length SDS	0.008	0.005, 0.011	<0.001	0.008	0.005, 0.011	<0.001
Crown–heel length Z-score SDS	0.004	0.001, 0.007	0.005	0.008	0.005, 0.012	<0.001
Head circumference SDS	−0.003	−0.006, 0.000	0.043	−0.004	−0.007, −0.001	0.009
Head circumference Z-score SDS	−0.006	−0.009, −0.003	<0.001	−0.004	−0.007, −0.001	0.008

^a^ Adjusted for maternal age, BMI, ethnicity, education, parity, anemia during pregnancy, occupation, folic use, follow-up age, SGA, infant sex, caesarean delivery, gestational age, and exclusive breastfeeding. Abbreviations: 95% CIs, 95% confidence intervals; SDS, standard deviation score; BMI, body mass index; SGA, small for gestational age.

**Table 4:** Stratified analysis of the effects of infant birth sizes on childhood anemia by maternal folic acid use and occupation.

Classifications	Birth Weight		Crown–Heel Length		Head Circumference	
	SDS	Z-Score SDS	SDS	Z-Score SDS	SDS	Z-Score SDS
Folic acid use						
Yes	−0.009 (−0.014, −0.004)	−0.008 (−0.013, −0.003)	0.010 (0.005, 0.014)	0.009 (0.005, 0.014)	−0.018 (−0.022, −0.014)	−0.019 (−0.023, −0.014)
No	−0.007 (−0.012, −0.002)	−0.006 (−0.011, −0.001)	0.007 (0.003, 0.011)	0.007 (0.003, 0.011)	0.012 (0.008, 0.016)	0.012 (0.008, 0.016)
*p*-value for interaction	<0.001		<0.001		<0.001	
Occupation						
Farmer	−0.008 (−0.013, −0.004)	−0.007 (−0.011, −0.002)	0.009 (0.005, 0.012)	0.009 (0.005, 0.012)	−0.007 (−0.011, −0.004)	−0.007 (−0.011, −0.004)
Non-farmer ^a^	−0.007 (−0.012, −0.001)	−0.007 (−0.013, −0.001)	0.007 (0.002, 0.013)	0.007 (0.001, 0.013)	0.004 (−0.001, 0.009)	0.004 (−0.002, 0.009)
*p*-value for interaction	<0.001		0.010		0.001	

The results are presented as β coefficients with 95% CIs in the multivariate regression models (adjusted for maternal age, BMI, ethnicity, education, parity, anemia during pregnancy, occupation, folic use, follow-up age, SGA, infant sex, caesarean delivery, gestational age, and exclusive breastfeeding). ^a^ Including workers who work in factories, businessperson, teacher, day laborer, civil servant, or unknown. Abbreviations: 95% CIs, 95% confidence intervals; SDS, standard deviation score; BMI, body mass index; SGA, small for gestational age.

## Data Availability

The data will be available upon reasonable request by contacting the corresponding author. The data are not publicly available due to privacy protection for the information of participants.

## Supplementary Materials

The following Supporting Information can be downloaded at: https://www.mdpi.com/article/10.3390/nu16121796/s1, Table S1: Descriptive analysis of peri/neonatal risk factors for childhood anemia.

## References

[1] Stevens G.A., Finucane M.M., De-Regil L.M., Paciorek C.J., Flaxman S.R., Branca F., Peña-Rosas J.P., Bhutta Z.A., Ezzati M. (2013). Global, regional, and national trends in haemoglobin concentration and prevalence of total and severe anaemia in children and pregnant and non-pregnant women for 1995–2011: A systematic analysis of population-representative data. Lancet Glob. Health.

[2] Kassebaum N.J., Jasrasaria R., Naghavi M., Wulf S.K., Johns N., Lozano R., Regan M., Weatherall D., Chou D.P., Eisele T.P. (2014). A systematic analysis of global anemia burden from 1990 to 2010. Blood.

[3] Chaparro C.M., Suchdev P.S. (2019). Anemia epidemiology, pathophysiology, and etiology in low- and middle-income countries. Ann. N. Y. Acad. Sci..

[4] Allali S., Brousse V., Sacri A.S., Chalumeau M., de Montalembert M. (2017). Anemia in children: Prevalence, causes, diagnostic work-up, and long-term consequences. Expert Rev. Hematol..

[5] Kassebaum N., Kyu H.H., Zoeckler L., Olsen H.E., Thomas K., Pinho C., Bhutta Z.A., Dandona L., Ferrari A., Ghiwot T.T. (2017). Child and Adolescent Health from 1990 to 2015: Findings from the Global Burden of Diseases, Injuries, and Risk Factors 2015 Study. JAMA Pediatr..

[6] Young M.F., Nguyen P., Tran L.M., Khuong L.Q., Martorell R., Ramakrishnan U. (2023). Long-Term Association between Maternal Preconception Hemoglobin Concentration, Anemia, and Child Health and Development in Vietnam. J. Nutr..

[7] Cordova E.G., Belfort M.B. (2020). Updates on Assessment and Monitoring of the Postnatal Growth of Preterm Infants. Neoreviews.

[8] Melve K.K., Gjessing H.K., Skjaerven R., Oyen N. (2000). Infants’ length at birth: An independent effect on perinatal mortality. Acta Obs. Gynecol. Scand.

[9] Aagaard K., Matthiesen N.B., Bach C.C., Larsen R.T., Henriksen T.B. (2020). Head circumference at birth and intellectual disability: A nationwide cohort study. Pediatr. Res..

[10] Piro E., Schierz I.A.M., Serra G., Puccio G., Giuffrè M., Corsello G. (2020). Growth patterns and associated risk factors of congenital malformations in twins. Ital. J. Pediatr..

[11] Widdowson E.M., Spray C.M. (1951). Chemical development in utero. Arch. Dis. Child..

[12] Chaparro C.M. (2008). Setting the stage for child health and development: Prevention of iron deficiency in early infancy. J. Nutr..

[13] Kc A., Rana N., Målqvist M., Jarawka Ranneberg L., Subedi K., Andersson O. (2017). Effects of Delayed Umbilical Cord Clamping vs. Early Clamping on Anemia in Infants at 8 and 12 Months: A Randomized Clinical Trial. JAMA Pediatr..

[14] Li N., An H., Jin M., Li Z., Zhang Y., Zhang L., Liu J., Ye R. (2022). Association of Infants Small for Gestational Age with Anemia under Five Years Old in Two Large Longitudinal Chinese Birth Cohorts. Nutrients.

[15] An H., Chen H., Li Z., Zhang L., Zhang Y., Liu J., Ye R., Li N. (2022). Association of Gestational Hypertension with Anemia under 5 Years Old: Two Large Longitudinal Chinese Birth Cohorts. Nutrients.

[16] Villar J., Carroli G., Wojdyla D., Abalos E., Giordano D., Ba’aqeel H., Farnot U., Bergsjø P., Bakketeig L., Lumbiganon P. (2006). Preeclampsia, gestational hypertension and intrauterine growth restriction, related or independent conditions?. Am. J. Obstet. Gynecol..

[17] Berry R.J., Li Z., Erickson J.D., Li S., Moore C.A., Wang H., Mulinare J., Zhao P., Wong L.Y., Gindler J. (1999). Prevention of neural-tube defects with folic acid in China. China-U.S. Collaborative Project for Neural Tube Defect Prevention. N. Engl. J. Med..

[18] Gindler J., Li Z., Berry R.J., Zheng J., Correa A., Sun X., Wong L., Cheng L., Erickson J.D., Wang Y. (2001). Folic acid supplements during pregnancy and risk of miscarriage. Lancet.

[19] Fok F.T. (2003). Updated gestational age specific birth weight, crown-heel length, and head circumference of Chinese newborns. Arch. Dis. Child. Fetal. Neonatal. Ed..

[20] WHO (2001). Iron deficiency anaemia: Assessment, prevention, and control. A Guide for Programme Managers.

[21] Marques P.C., Rocha G., Flor D.E.L.F., Guimarães H. (2022). Extrauterine growth restriction at discharge in very-low-birth weight infants: A retrospective study in a level III neonatal intensive care unit. Minerva Pediatr..

[22] Yang W., Li X., Li Y., Zhang S., Liu L., Wang X., Li W. (2012). Anemia, malnutrition and their correlations with socio-demographic characteristics and feeding practices among infants aged 0–18 months in rural areas of Shaanxi province in northwestern China: A cross-sectional study. BMC Public Health.

[23] Chandran V., Kirby R.S. (2021). An Analysis of Maternal, Social and Household Factors Associated with Childhood Anemia. Int. J. Environ. Res. Public Health.

[24] Amrutha Veena K., Kowsalya S., Kothandapani S. (2014). Micronutrient malnutrition profile of infants in South India. J. Hum. Nutr. Diet..

[25] Lander R.L., Enkhjargal T., Batjargal J., Bailey K.B., Diouf S., Green T.J., Skeaff C.M., Gibson R.S. (2008). Multiple micronutrient deficiencies persist during early childhood in Mongolia. Asia Pac. J. Clin. Nutr..

[26] Wong A.Y., Chan E.W., Chui C.S., Sutcliffe A.G., Wong I.C. (2014). The phenomenon of micronutrient deficiency among children in China: A systematic review of the literature. Public Health Nutr..

[27] An H., Liu X., Li Z., Zhang L., Zhang Y., Liu J., Ye R., Li N. (2023). Association of age at menarche with gestational hypertension and preeclampsia: A large prospective cohort in China. J. Clin. Hypertens.

[28] Lopez A., Cacoub P., Macdougall I.C., Peyrin-Biroulet L. (2016). Iron deficiency anaemia. Lancet.

[29] McCarthy E.K., ni Chaoimh C., Kenny L.C., Hourihane J.O., Irvine A.D., Murray D.M., Kiely M.E. (2018). Iron status, body size, and growth in the first 2 years of life. Matern. Child Nutr..

[30] Marques O., Weiss G., Muckenthaler M.U. (2022). The role of iron in chronic inflammatory diseases: From mechanisms to treatment options in anemia of inflammation. Blood.

[31] Ragsdale H.B., Kuzawa C.W., Borja J.B., Avila J.L., McDade T.W. (2019). Regulation of inflammation during gestation and birth outcomes: Inflammatory cytokine balance predicts birth weight and length. Am. J. Hum. Biol..

[32] Koirala J., Raddi S.A., Dalal A.D. (2022). Maternal Anemia and BMI as Determinants of Pregnancy Outcomes: A Hospital-Based Study. J. Nepal. Health Res. Counc..

[33] Hauta-Alus H.H., Viljakainen H.T., Holmlund-Suila E.M., Enlund-Cerullo M., Rosendahl J., Valkama S.M., Helve O.M., Hytinantti T.K., Mäkitie O.M., Andersson S. (2017). Maternal vitamin D status, gestational diabetes and infant birth size. BMC Pregnancy Childbirth.

[34] Abioye A.I., McDonald E.A., Park S., Ripp K., Bennett B., Wu H.W., Pond-Tor S., Sagliba M.J., Amoylen A.J., Baltazar P.I. (2019). Maternal anemia type during pregnancy is associated with anemia risk among offspring during infancy. Pediatr. Res..

[35] Yang G., Fan L., Tan J., Qi G., Zhang Y., Samet J.M., Taylor C.E., Becker K., Xu J. (1999). Smoking in China: Findings of the 1996 National Prevalence Survey. JAMA.

[36] Silver M.K., Arain A.L., Shao J., Chen M., Xia Y., Lozoff B., Meeker J.D. (2018). Distribution and predictors of 20 toxic and essential metals in the umbilical cord blood of Chinese newborns. Chemosphere.

[37] Shah F., Kazi T.G., Afridi H.I., Baig J.A., Khan S., Kolachi N.F., Wadhwa S.K., Shah A.Q. (2010). Environmental exposure of lead and iron deficit anemia in children age ranged 1-5years: A cross sectional study. Sci. Total Environ..

[38] Csoelle I., Felso R., Szabo E., Metzendorf M.I., Schwingshackl L., Ferenci T., Lohner S. (2022). Health outcomes associated with micronutrient-fortified complementary foods in infants and young children aged 6–23 months: A systematic review and meta-analysis. Lancet. Child Adolesc. Health.

[39] Liu J.M., Li Z., Lin Q., Wang T.M., Hong S.X., Zheng J.C., Ji C.Y., Zhao F.L., Li S. (2001). Intrauterine growth retardation and cerebral palsy. Chin. J. Pre. Med..

