# Supplementary material for: The Association of Infant Birth Sizes and Anemia under Five Years Old: A Population-Based Prospective Cohort Study in China
Source: Nutrients. 2024 Jun 7;16(12):1796. doi: 10.3390/nu16121796 (PMC11206821; doi:10.3390/nu16121796)
Supplement: Supplementary file 1 [file nutrients-16-01796-s001.zip › nutrients-3004302-supplementary.pdf]

Table S1. Descriptive analysis of peri/neonatal risk factors for childhood anemia.

	Total participants (N=204,556)		Frequency (rate, %)
	Missing	Valid	
Infant sex	226	204,330	
Male			106,260 (52.0)
Female			98,070 (48.0)
Delivery mode	0	204,556	
Caesarean section			38,870 (19.0)
Others			165,686 (81.0)
SGA	66	204,490	10,848 (5.3)
IUGR	4559	199,997	287 (0.1)

Abbreviations: SGA, small for gestational age; IUGR, intrauterine growth retardation.
